# Human α-Galactosidase A Mutants: Priceless Tools to Develop Novel Therapies for Fabry Disease

**DOI:** 10.3390/ijms22126518

**Published:** 2021-06-17

**Authors:** Andrea Modrego, Marilla Amaranto, Agustina Godino, Rosa Mendoza, José Luis Barra, José Luis Corchero

**Affiliations:** 1 Institut de Biotecnologia i de Biomedicina, Universitat Autònoma de Barcelona, Bellaterra, 08193 Barcelona, Spain; modrego82@gmail.com (A.M.); rmendoza@ciber-bbn.es (R.M.); 2 Centro Nacional de Biotecnología, Consejo Superior de Investigaciones Científicas (CSIC), 28049 Madrid, Spain; 3 Departamento de Química Biológica Ranwel Caputto, Centro de Investigaciones en Química Biológica de Córdoba, CONICET, Facultad de Ciencias Químicas, Universidad Nacional de Córdoba, Córdoba 5016, Argentina; marilla.amaranto@gmail.com (M.A.); agustinagodino@gmail.com (A.G.); jlbarra@fcq.unc.edu.ar (J.L.B.); 4 Centro de Investigación Biomédica en Red en Bioingeniería, Biomateriales y Nanomedicina (CIBER-BBN), c/Monforte de Lemos 3–5, 28029 Madrid, Spain; 5 Departament de Genètica i de Microbiologia, Universitat Autònoma de Barcelona, Bellaterra, 08193 Barcelona, Spain

**Keywords:** alpha-galactosidase A, Fabry disease, pharmacological chaperones, rare diseases, enzyme replacement therapy

## Abstract

Fabry disease (FD) is a lysosomal storage disease caused by mutations in the gene for the α-galactosidase A (GLA) enzyme. The absence of the enzyme or its activity results in the accumulation of glycosphingolipids, mainly globotriaosylceramide (Gb3), in different tissues, leading to a wide range of clinical manifestations. More than 1000 natural variants have been described in the GLA gene, most of them affecting proper protein folding and enzymatic activity. Currently, FD is treated by enzyme replacement therapy (ERT) or pharmacological chaperone therapy (PCT). However, as both approaches show specific drawbacks, new strategies (such as new forms of ERT, organ/cell transplant, substrate reduction therapy, or gene therapy) are under extensive study. In this review, we summarize GLA mutants described so far and discuss their putative application for the development of novel drugs for the treatment of FD. Unfavorable mutants with lower activities and stabilities than wild-type enzymes could serve as tools for the development of new pharmacological chaperones. On the other hand, GLA mutants showing improved enzymatic activity have been identified and produced in vitro. Such mutants could overcome several complications associated with current ERT, as lower-dose infusions of these mutants could achieve a therapeutic effect equivalent to that of the wild-type enzyme.

## 1. Fabry Disease

Fabry disease (FD) is an X-linked inherited lysosomal storage disease (LSD). The cause of the disease is the mutation of the gene that encodes the enzyme α-galactosidase A (GLA), which leads to a total or partial loss of its function. GLA is a lysosomal enzyme that belongs to the glycoside hydrolase family, and it is responsible for hydrolyzing alpha-galactosyl terminal groups of glycolipids and glycoproteins. Its main substrate is globotriaosylceramide (Gb3), a glycosphingolipid consisting of a ceramide attached to a sugar chain of one glucose and two galactoses. When Gb3 is not properly metabolized, it mainly accumulates in the lysosomes, endothelial cells being one of the most affected cell types. As a result, different signs and symptoms associated with the disease begin to manifest [1]. Other minor substrates of GLA include a deacylated form of Gb3, called globotriaosylsphingosine (lyso-Gb3), digalactosylceramide, and blood group B glycosphingolipids.

FD is considered an attenuated disease as patients survive to adulthood. However, those who lack GLA activity completely have a shortened life expectancy. FD patients present a wide and diverse spectrum of clinical manifestations, varying from classic FD in males to asymptomatic disease in females, with several variants between these two extremes [2]. Currently, more than 1000 different mutations have been described [3], many of which are “private” as they only occur in one family, resulting in a high heterogeneity of the disease. Moreover, there is a high phenotypic variability even between individuals with the same pathogenic variant, which makes it very difficult to establish genotype-phenotype correlations. Having only one X chromosome, males are hemizygous for the pathogenic mutation, so they generally develop more severe signs and symptoms. As a result, the most severe phenotype or “classic phenotype” occurs predominantly in males and is characterized by zero or minimal residual activity of the enzyme. However, unlike other X-linked disorders, females may experience significant symptoms of the disease depending on their residual GLA activity. Clinical manifestations are present in two thirds of women, who generally suffer from a milder disease. This heterogeneity is explained by the process of lyonization, which consists of the random inactivation of one of the X chromosomes in the tissues and organs of the female [4].

Patients with the classic form of FD show no residual enzyme activity [5]. However, the residual activity threshold resulting in FD has not been clearly established. In this sense, it has been estimated that a residual activity of 30–35% of the mean normal α-galactosidase A activity is the cut-off to diagnose Fabry disease [6]. Moreover, such a threshold may vary between organs and even between patients. The nonspecific, multi-organic nature and the relatively high population frequency of FD are risks for the wrong attribution of pathogenicity to certain GLA mutants. This can lead to unnecessary, expensive, and invasive therapies such as ERT. Therefore, before concluding that a mutant variant is pathogenic, one must demonstrate evidence of altered sphingolipid homeostasis. Clinical symptoms are mild at first, which can make diagnosis difficult or delayed, especially if there is no previous family history [7]. Symptoms are usually progressive and affect multiple organs. However, due to the heterogeneity of the disease, there are patients who experience phenotypes in just a single organ (for example, cardiac variant). This occurs mainly in those patients with significant residual activity [1]. Among the characteristic symptoms of FD, there are neurological (pain), skin (angiokeratoma), kidney (proteinuria and kidney failure), cardiovascular (cardiomyopathy and arrhythmia), cochlear-vestibular, ocular, and cerebrovascular problems (transient ischemic attacks and strokes). In addition, anhidrosis or hypohidrosis can occur, causing intolerance to heat and exercise [1].

FD presents a complicated diagnosis, particularly in childhood, as the symptoms are often nonspecific, and the disease is not widely known [8]. Furthermore, some of the characteristic symptoms such as kidney and heart dysfunction appear only in more advanced stages of the disease. In males, hemizygous patients, enzyme activity can be used for diagnosis as it is drastically reduced or does not exist. For this, GLA activity in leukocytes is measured, and if it is significantly lower than normal values, FD can be diagnosed. However, in female patients and some variants of males, it is common that false negatives occur as activity can vary considerably in different cell and tissue types. Therefore, to diagnose a female, the most reliable method is the sequencing of the GLA gene [9]. This test can detect a disease-causing mutation in more than 97% of patients [2], but it has a high cost, making it difficult to use widely. Many attempts have been made over the years to find an ideal disease-specific marker that could serve as a rapid screening tool, as well as an indicator of response to the treatment. Gb3 was initially tested as a possible marker of disease; however, some healthy women showed elevated Gb3 levels in urine, making it difficult to differentiate FD patients from healthy ones. On the other hand, it was found that the levels of lyso-Gb3 (product of Gb3 degradation, as well as minor substrate of GLA) correlate with the clinical condition and the type of mutation, thus making it a reliable predictor of the disease [2]. Furthermore, various studies have demonstrated that the levels of lyso-Gb3 decrease in patients undergoing ERT, particularly in patients with the classic phenotype [10]. However, even lyso-Gb3 has proven not to be always reliable as a FD biomarker. For example, a recent study by Bichet et al. showed that lyso-Gb3 is not a prognostic biomarker of migalastat treatment response in patients with FD [11], a finding similar to another one published for ERT [12]. These findings would compromise the usefulness of lyso-Gb3 as a biomarker in treatment follow-up. In this context, other possible markers (microRNAs, new Gb3 isoforms, and abnormal protein excretion) have been studied but are not yet widely used due to lack of data [13,14,15].

## 2. The Human Gla Enzyme

### 2.1. Structure of GLA Enzyme

The GLA gene encoding the GLA enzyme is located in the long arm of the X chromosome at position Xq22, spans approximately 13 kb, and contains seven exons. The cDNA contains 1290 bases and, upon translation, results in a protein of 429 amino acids that include a 31 amino acid signal peptide [16].

In lysosomes, the glycoprotein is presented as a homodimer, in which each monomer contains two domains. The N-terminal domain spans from residue 32 to 330 and is a classic (β/α)8 barrel with the active site in its center. The C-terminal domain extends from residue 331 to 429 and consists of an antiparallel β domain. Each monomer contains three N-linked carbohydrate sites, five disulfide bonds, two unpaired cysteine residues, and three cis proline residues (P210, P380, and P389) [17].

There are four potential N-glycosylation sites (N139, N192, N215, and N408), but only the first three (located in the first domain) are occupied by oligosaccharides. Of these, the third site N215 is significantly important for the solubility of the enzyme, as well as for its transport from the endoplasmic reticulum to the lysosomes [18]. In the structural analysis of the oligosaccharides linked to the glycosylation sites, it is observed that the oligosaccharides of the secreted enzyme are markedly heterogeneous, with structures containing high levels of mannose, as well as complex or hybrid structures. Furthermore, a significant number of the oligosaccharides have phosphate monoester groups [19]. The absorption of the enzyme in the lysosomes is mediated by mannose-6-phosphate receptors (MPRs), which explains the presence of high levels of mannose-6-phosphate.

Each GLA monomer contains 12 cysteine residues. Ten of them form disulfide bonds (C52-C94, C56-C63, C142-C172, C202-C223, and C378-C382) within the monomeric subunits, while the remaining two (C90 and C174) contain free sulfhydryl groups. Disulfide bonds play important roles for protein structure and stability. Furthermore, cysteine C142 is part of the active site of the enzyme, which is why the disulfide bond C142-C172 is relevant for the enzymatic activity. On the other hand, free cysteines play important roles in both protein structure and function, including dimerization, enzyme catalysis, redox regulation, and thermal stability [20]. The two free cysteines in GLA are found in different structural environments within the folded protein, which modulates the function of each one within it [21]. C90 is completely buried within the structure, while C174 is partially exposed on the surface of the protein. The active site is formed by the side chains of residues W47, D92, D93, Y134, C142, K168, D170, E203, L206, Y207, R227, D231, D266, and M267, with C172 making a disulfide bond with C142.

### 2.2. GLA Synthesis and Trafficking

The GLA enzyme precursor is synthesized in the rough endoplasmic reticulum and modified in the Golgi apparatus, where newly synthesized GLA acquires mannose-6-phosphate (M6P) moieties on its N-linked oligosaccharidic chains. These M6P residues serve as recognition signals for mannose-6-phosphate receptors (M6PRs) that transport GLA to endolysosomes in clathrin-coated vesicles through a cation-dependent (CD) and a cation-independent (CI) pathway. While the CD-M6PR appears to act intracellularly (i.e., in the trans-Golgi network, TGN, to endosome sorting), the CI-M6PR can mediate GLA transport from the TGN, as well as from the cell surface, helping to recapture secreted GLA. Once in the endosomes, the GLA-M6PR complex dissociates in the acidic environment, and the receptors recycle to their compartments of origin (plasma membrane and/or TGN) [22].

### 2.3. Catalytic Mechanism of GLA

The GLA enzyme is an α-retention glycoside hydrolase, because both the substrate and the product have anomeric carbons with α configurations. The mechanism of these enzymes occurs by means of a double displacement reaction, where two consecutive nucleophilic attacks on the anomeric carbon lead to the general retention of the anomeric configuration. Two carboxylates are required, one acting as a nucleophile and the other as an acid/base [23]. The first nucleophilic attack on the substrate comes from D170, which cleaves the glycosidic bond and results in the formation of a covalent enzyme-intermediate bond. In the second step of the reaction, a molecule of water (deprotonated by D231) attacks carbon 1 of the covalent intermediate, releasing the second half of the catalytic product and regenerating the enzyme to its initial state. The enzyme is more effective at low pH, according to its highly acidic composition and lysosomal location [24].

## 3. Current Treatments For FD

There have been numerous advances in the knowledge of the disease pathogenesis, as well as in its treatment. However, current available therapies are not curative, as they only delay the progression of the disease, in addition to presenting several limitations. Currently, two therapeutic approaches are approved for FD treatment: enzyme replacement therapy (ERT) and pharmacological chaperone therapy (PCT).

### 3.1. Enzyme Replacement Therapy

ERT consists of systemic infusions of recombinant GLA. In this way, it is possible to replace the enzyme that is absent or deficient and eliminate, or reduce, the accumulation of Gb3 [25]. Treatment should be started as soon as the diagnosis is made, regardless of whether there are clinical manifestations or not, as Gb3 deposits already begin in intrauterine life [2]. Two formulations of recombinant human GLA have been developed: alpha agalsidase (Replagal, by Shire) and beta agalsidase (Fabrazyme, by Sanofi Genzyme). The first one is produced by overexpression in human fibroblasts and the second one in CHO cells. They are administered by intravenous infusions every two weeks (agalsidase alpha at a dose of 0.2 mg/kg and agalsidase beta at a dose of 1 mg/kg). Both treatments result in the reduction of Gb3 plasma and urinary levels, as well as decreased storage of glycosphingolipids in lysosomes. The ERT response in patients can be influenced by several factors, an important one being the formation of anti-drug antibodies (ADAs). A high percentage of patients with Fabry disease lack the GLA enzyme completely; therefore, when the recombinant enzyme is administered, the immune system recognizes it as nonself, triggering an immune response. The apparition of ADAs may negatively influence the long-term efficacy of the treatment [26].

Apart from ADA formation, ERT for FD presents other potential limitations. It has a limited tissue penetration and lacks efficacy if started at advanced stages of the disease. GLA does not pass the blood–brain barrier and it may induce infusion adverse reactions. Finally, ERT is a lifelong therapy requiring intravenous administration every 2 weeks, where it is also associated with a high cost of the treatment.

### 3.2. Pharmacological Chaperones

On the other hand, PCT is based on the use of small molecules (pharmacological chaperones, PCs) capable of stabilizing the mutant enzyme to ensure its correct folding. This prevents the degradation of the enzyme by unproper folding, allowing trafficking to the lysosomes where the PC and mutant enzyme dissociate, so that the enzyme can exert its function. The term “pharmacological chaperones” appears to label those molecules that can act as drugs, facilitating the proper folding of certain mutant proteins with incorrect folding. PCs should not be mistaken for molecular chaperones, as they are low-molecular-weight chemical molecules instead of proteins [27].

Lysosomal storage diseases have become an important target in the study and development of PCs, as the ER quality control often recognizes mutant forms of lysosomal enzymes that present minor modifications in their stability or conformation, but still retain catalytic activity. It results in a premature enzyme degradation, which prevents trafficking through the secretory pathway and causes the loss of function.

For PCT to be effective, patients must have susceptible (amenable) mutations, that is, those with certain GLA residual activity [28]. Currently, there are at least 367 amenable and 711 nonamenable mutations known, based on an in vitro assay [29]. The first studies on PCT were carried out for FD. In 1995, a study carried out by Okumya et al. [30] showed that the administration of galactose stabilized GLA mutants, resulting in a higher secretion and activity. Later, in 1999, a galactose analog (an iminosugar called 1-deoxygalactonojirimycin, DGJ) was found to be more efficient than galactose in stabilizing GLA mutants [31]. DGJ binds reversibly and selectively to the active site of GLA with high affinity. Iminosugars such as DGJ are charged molecules with a low capacity to cross membranes due to a negative octanol-water partition coefficient. Modifications to enhance lipophilicity with alkylation have been proposed but resulted in lowered DGJ efficacy [32]. DGJ is a potent GLA inhibitor, stabilizing the enzyme at both neutral and acidic pH. This means that it can stabilize the enzyme in the ER, but it can also cause a partial inhibition of the enzyme in the lysosome [33]. Due to this, for DGJ to be effective, its administration has to be intermittent as the half-life of most GLA mutants is longer than the half-life of DGJ in vivo. Nowadays, PCT with DGJ is approved for FD treatment in patients with amenable mutations under the name “Migalastat,” a drug developed by Amicus Therapeutics.

For amenable mutations resulting in the synthesis of a defective enzyme in which activity and stability are altered, PCT by orally administered Migalastat might be a better choice for addressing certain unmet medical needs associated with ERT (like antibody formation). On the other hand, inhibitors are not the ideal drugs, as they may not be able to fully revert the phenotype of the disease. As seen, DGJ represents a good starting point but has several limitations that could be improved. Therefore, the study of novel PCs could overcome this limitation, increasing therapy effectiveness. Due to this, it has been proposed to modify first-generation PCs to enhance their therapeutic effects. To test these (and others) new potential PCs, the identification of amenable mutations and, ideally, their production as recombinant enzymes is needed.

## 4. GLA Mutations

Currently, more than 1000 different mutations have been described in the GLA gene. According to the Human Gene Mutation Database [3], such mutations can be sorted in different categories: missense/nonsense (~69%), deletions (~19%), insertions (~6%), splicing defects (~5%), and others (~2%). They are related to several abnormalities, such as irregular intracellular trafficking, altered protein folding, reduced activity of the active site, and lower affinity for substrates.

### 4.1. Most Frequent GLA Mutant Variants

Isolation and sequencing of the entire genomic sequence of the GLA gene allowed the detection and characterization of the mutations causing FD. Furthermore, diffusion of screening studies in the high-risk population and in newborns has contributed to the identification of numerous genetic variants. Among the most frequent variants described in the GLA gene, there are E66Q, A143T, D313Y, R118C, N215S, M296I, and R112H [4]. However, a percentage of mutations are classified by the scientific community as “genetic variants of unknown significance” (GVUS), as it cannot be stated with certainty whether they are benign or pathogenic [34].

The classic phenotype in males can be caused by a diversity of GLA variants, including genetic rearrangements of different sizes, splicing defects, and missense/nonsense mutations [35]. On the other hand, people with atypical late-onset variants (kidney, cardiac, or cerebrovascular disease) present mainly missense and splicing mutations that lead to residual enzymatic activity. However, several pathogenic variants (e.g., R301Q) have been identified to appear both in individuals with the classic phenotype and cardiac variant, indicating that there may be other important factors in the manifestation of the disease [36]. Due to this, the correlation between genotype and phenotype is complex as the same mutation can give rise to different clinical manifestations.

### 4.2. Most Frequently Mutated Residues

Mutations in the GLA gene can be found throughout the whole sequence. According to the Human Gene Mutation Database, 69% of the residues of GLA have been found to be mutated in patients with Fabry disease. There are certain amino acids in which mutations are most frequently located. Tryptophan is the amino acid with the highest mutation frequency in the GLA-described mutations. Of the 17 residues of tryptophan within the protein, 15 of them are found mutated in patients with Fabry disease (94% mutation rate). They are usually nonsense mutations, which can negatively affect the internal folding of the protein as tryptophan contributes to the formation of the hydrophobic nucleus.

The second most frequently mutated residue is cysteine. Of the 13 cysteines present in the protein, only the cysteine of the signal peptide has not been found in any patient with Fabry disease, which gives a mutation rate of 92%. Numerous mutations in cysteines involved in disulfide bond formation have been identified, while mutations in free cysteines are less common. Mutations in cysteine residues that form disulfide bonds result in incorrect folding of the protein and its complete or partial lack of function, indicating its importance in protein folding and thermodynamic stability. In most of the cases described, mutations affecting disulfide bonds lead to the severe form of Fabry disease [37]. Mutations in free cysteines (C90X, C174G, and C174R) have been described but appear much less frequently and are associated with a late, attenuated phenotype of the disease [38,39,40]. Consequently, free cysteine residues play a minor structural role within the protein versus residues that form disulfide bonds. Studies of recombinant GLA have revealed the tolerance of free cysteine residues to amino acid change [37]. C90 is completely buried inside the protein surrounded by relatively large hydrophobic and aromatic residues (L45, Y88, L166, and F295) and covered by K168. Therefore, this position only tolerates certain conservative mutations with small side chains such as C90S, C90A, C90T, and C90V. In the case of C174, the amino acid change causes loss of activity, indicating a greater importance in the protein. This residue is found on the surface of the enzyme with the sulfhydryl side chain partially buried. Moreover, it is close to the disulfide bond formed by C142 and C172, which is part of the active site. This explains the greater impact of mutations in this position compared to the other free cysteine. Other frequently mutated residues are glycine and arginine. As glycine is the smallest amino acid, changes to more voluminous amino acids could result in an alteration in the stability of the protein. Glycine has a mutation rate of 84% in described mutations, as, of the 33 residues of glycine, 28 have a described mutation in the Human Gene Mutation Database. Arginine can establish both hydrophobic and hydrophilic interactions. There are 21 residues of arginine within the sequence of the protein and 13 are found on the list of point mutations (62% mutation rate). Lysine is chemically similar to arginine but has a mutation rate significantly lower (35%), indicating the importance of arginine in the functioning of the enzyme. The explanation could be that arginine is buried or partially buried inside the protein more frequently than lysine, which is almost always exposed on the surface. Other residues commonly mutated are those that are part of the active site of the protein or are close to it. All residues that participate in the catalytic activity of the protein have been described with mutations in patients with FD; therefore, the active center is highly sensitive to mutations [41]. In the case of glycosylation sites, the main site affected by mutations that give rise to disease phenotypes is N215. However, different clinical manifestations have been described for the same mutation (N215S), including moderate phenotype [42], attenuated [43], and atypical variants [44]. This site, as previously mentioned, is important for the enzyme’s solubility, as well as for its transport from the endoplasmic reticulum to the lysosomes, which explains the appearance of the disease. Mutations at this site would alter the correct folding and transport of GLA, resulting in a nonfunctional protein [45]. The presence of an adjacent hydrophobic sequence supports the importance of this site in the solubility of the enzyme, as the hydrophobic zone must be covered by the oligosaccharide to prevent the aggregation and degradation of the enzyme.

### 4.3. Nonsense Mutations

Finally, nonsense mutations, where the mutation produces a stop codon, are also common. Premature termination results in shortened proteins at the C-terminus, and sometimes, the protein cannot be produced, due to early degradation of the mRNA. Generally, this type of mutation is associated with a severe phenotype. However, removal of the first residues has been found to give rise to a protein with superior enzyme activity [46,47].

### 4.4. Location-Dependent Biological Effect of Mutations

**Figure 1:**
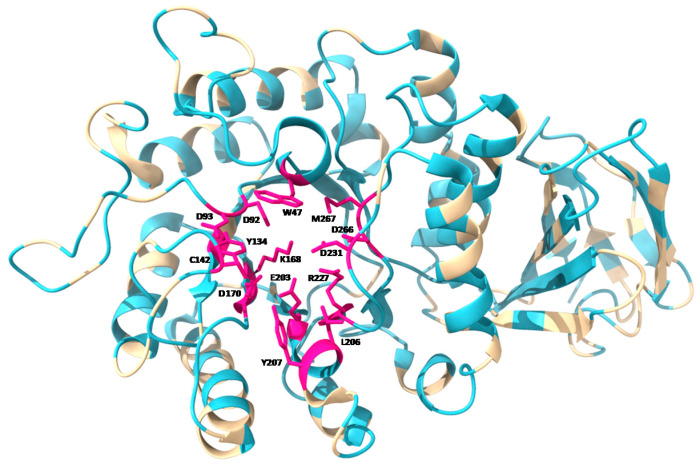
Tertiary structure of GLA monomer, showing (in blue) the location of point mutations described according to Human Gene Mutation Database [3], as well as the residues involved in the active site of the enzyme (in pink). Mutations for all residues in the active site have been found and described. Image obtained using UCSF ChimeraX software, based on PDB: 1R46 structure.

Mutations in different locations of the protein can cause different effects. Mutations in enzyme active site residues result in a significant or total loss of enzymatic activity, while mutations in hidden residues inside the protein hamper the formation of the hydrophobic nucleus and affect correct folding. Both types of alterations can lead to the clinical manifestation of the disease. Figure 1 shows the location of described mutations throughout the GLA monomer. Mutations in the proximity of the active site are common. However, most of the residues affected by point mutations in FD are distributed throughout the protein sequence and, mainly, in the hydrophobic nucleus, where more than half of the described mutations are found [48]. These mutations, which do not directly affect the active site of the protein, often lead to proteins that retain some residual activity. Residues affecting the correct folding of the protein have been found throughout the full-length protein, while mutations directly affecting the activity of the protein occur preferentially within, or in close proximity to, the active site [49].

### 4.5. GLA Mutants with Low Activity and/or Stability

The majority of the above-mentioned GLA mutations are disease-causing. Taking into account the mechanism of pathogenesis, mutations can be divided into two groups: mutations that directly affect the active site (“catalytic”) and mutations that affect the correct folding of the protein (“noncatalytic”). Between these two, “noncatalytic” mutations are more prevalent, a fact that leads to the conclusion that FD is mainly a protein folding disease [1]. Several studies analyzing the activity and stability of these mutants confirmed that mutations interfering with the correct folding lead to proteins with residual activity and that are prone to degradation. This results in a loss of function, as they are not able to escape the ER and travel to the lysosome [28]. Further characterization of the mutants will allow the development of improved treatments for the disease, particularly in the case of mutations concerning the structural conformation of the protein.

#### Mutants as Tools to Develop Pharmacological Chaperones

Pathogenic mutants affecting correct GLA folding are excellent tools to study and develop the previously mentioned PCT. The current PC used to treat patients with amenable mutations of GLA is DGJ, which represents a good starting point but has several limitations that could be improved. In this context, mutants with improper folding produced in vitro could have an important application used as a tool for the development of new PCs.

**Figure 2:**
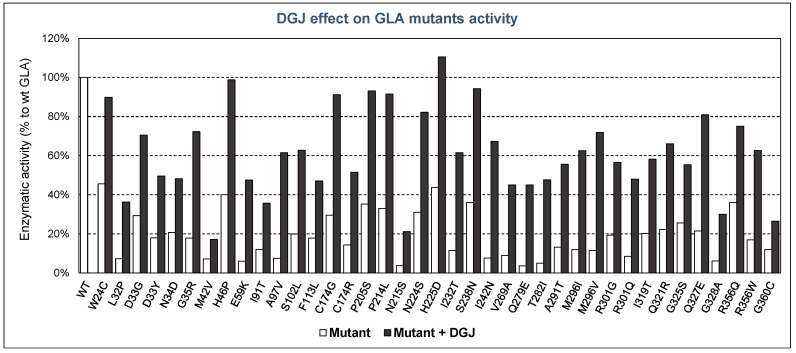
Enzymatic activity of GLA mutants compared with that of wild-type enzyme, and effect of DGJ addition. In white, residual enzymatic activity without DGJ; in black, enzymatic activity after adding DGJ. The mutants shown are examples described by several authors.

The residual activity and stability of GLA mutants have been measured in the presence or absence of pharmacological chaperones, mainly DGJ, by several authors, demonstrating the efficacy of this type of therapy in the treatment of FD. In Figure 2, some examples of known mutants with decreased activity are summarized, showing their enzymatic activity (in respect of that of the WT enzyme) both with and without DGJ.

As can be seen, in all the GLA mutants tested, DGJ had a positive effect on the enzymatic activity displayed by such mutants. In some cases, enzymatic activity reached values very close (or even higher) compared to the wild-type enzyme. Moreover, in vitro prediction of the efficacy of PCs is possible, as the responses obtained by recombinant mutants of GLA have shown a high degree of consistency with the responses of Fabry patient’s cells, as indicated in several studies [49,50]. Thus, it can be concluded that these DGJ in vitro tests are a reliable tool to measure the residual activity ex vivo and responsiveness to PCs.

Derivates of DGJ with improved therapeutic effect could also be examined. For example, Yu et al. synthetized a DGJ-thiourea derivate with high efficacy to enhance the residual activity of FD-associated GLA mutants, reducing the accumulation of the substrate Gb3 in FD cells. This chaperone, although inhibiting GLA at both neutral and acidic pH, showed a superior chaperoning effect than DGJ [51]. To avoid the inhibition of the enzyme in the lysosome, pH-sensitive derivates have been proposed. The strategy suggested by Mena-Barragán et al. [52] is based on the incorporation of an orthoester segment into the iminosugar conjugate to switch the hydrophobic-hydrophilic balance of the molecule on going from the neutral ER to the acidic lysosome. This has a dramatic effect on the enzyme binding affinity, leading to irreversible dissociation of the PC-enzyme complex at the lysosome [52].

Although pH-responsive chaperones could correct the problem of lysosomal inhibition, the ideal PC would be an allosteric ligand in order to achieve stabilization of missense mutations without obstructing the active site of the enzyme.

In addition to modifying first-generation PCs, another option to search for new potential molecules with these characteristics is molecular docking. Citro et al. identified an allosteric hot-spot for ligand binding by in silico docking, where 2,6-dithiopurine (DTP), a drug-like compound also identified in the study, binds preferentially. DTP showed a stabilization of lysosomal GLA in vitro also in mutants that do not show responsiveness to DGJ [53].

Another interesting approach in the search for new molecules for PCT is “drug repositioning,” which consists of using existing drugs for new therapeutic approaches. This strategy is particularly interesting for neglected diseases as it can speed up the development of the drug and lower the costs [54]. In this way, the screening of FDA-approved drugs could result in the discovery of novel molecules with the ability of assist lysosomal enzymes, such as GLA, to achieve the proper folding. As a matter of fact, there are several cases in which small, approved drugs have been successfully repositioned as PCs for rare diseases. An example is Diltiazem, an antihypertensive, that produces the restoration of mutant glucocerebrosidase activity in cells from patients with Gaucher disease [55]. In the case of FD, it was reported that Rosiglitazone (RSG), an antidiabetic approved by the FDA, when used in monotherapy or in conjunction with DGJ, resulted in a significant enhancement of mutant GLA activity [56].

Another small molecule identified through high-throughput screening is Ambroxol, an expectorant approved by the FDA. Ambroxol was found to be an inhibitor and stabilizer of lysosomal acid glucosylceramidase (Gaucher disease). However, its affinity is much lower in the acidic pH of the lysosome, allowing its dissociation [57]. Ambroxol has also showed efficacy for lysosomal GLA, but only in combination with DGJ [56]. This indicates the possibility of finding drugs that could act in different lysosomal storage diseases. The explanation for the wide-ranging effect of Ambroxol could be explained by the structural similarity of lysosomal enzymes, as well as the possible triggering of other stabilizing mechanisms apart from binding the enzyme.

There are other small molecules that can stabilize missense mutants, such as proteostasis regulators (PRs), which enhance the capacity of the proteostasis network [58]. PRs can activate the protein quality control of the cell so that the availability of molecular chaperones is increased. Additionally, they can directly enhance the function and activity of chaperones. This results in reduced protein misfolding due to a high protein folding capacity [59]. PRs were first tested as therapeutic drugs to treat lysosomal storage diseases. Seemann et al. investigated PRs for the treatment of FD, which resulted in the identification of small molecules (proteasome inhibitors such as MG132, BTZ, and CLC and an inhibitor of the ERAD) able to increase mutant GLA activity in patient-derived fibroblasts [60]. PRs can also work together with PCs in a synergistic mode of action to revert the disease of the phenotype, potentiating their therapeutic effect [61]. In the case of FD, the previously commented PRs identified also acted synergistically with DGJ, demonstrating the potential of combination treatment in a therapeutic application [60].

As already mentioned, not all GLA mutations can be corrected by PCT. Only patients with specific responsive, amenable mutations (those affecting correct protein folding and with residual activity) can benefit from this treatment [28]. Most GLA mutations leading to the manifestation of FD are private mutations, which makes it difficult to estimate the rate of responsive mutations. Due to this, further characterization of each described mutation is needed to determine which mutations are responsive and which are not. As different laboratories have developed their own assays to investigate lysosomal glycosidases, assay parameters (and conclusions) are often controversial. Therefore, a universal assay to check the responsiveness to PCT is needed. In any case, the current definition for a responsive mutation is with increases of 20% in the relative enzyme activity and 5% in the absolute enzyme activity compared to the wild-type enzyme [62].

There is a database, FabryCEP, that provides the comparison of results obtained by different experimental approaches for hundreds of GLA mutants in response to DGJ [63]. Additionally, if there are no experimental data for a specific mutation, it incorporates a predictive tool that provides a probability of a given mutation to be responsive to the drug based on a structural, functional, and evolutionary analysis.

Altogether, there are multiple ways in which PCT can be improved for the treatment of FD. In any case, the identification, production, characterization, and testing of unfavorable GLA mutants to test the efficacy of potential drugs with therapeutic effect are required.

### 4.6. GLA Mutants with Improved Activity and/or Stability

As previously described, most of the GLA mutations have been found in patients suffering from FD. However, mutations leading to a GLA with improved properties such as activity, stability, and/or secretion than the wild-type enzyme have also been described. Such mutations are not detected, as people expressing these “improved mutants” are expected to be phenotypically healthy and do not develop FD. However, different works involving the production of recombinant GLA mutants have found and described mutations rendering improved versions of the GLA enzyme.

**Figure 3:**
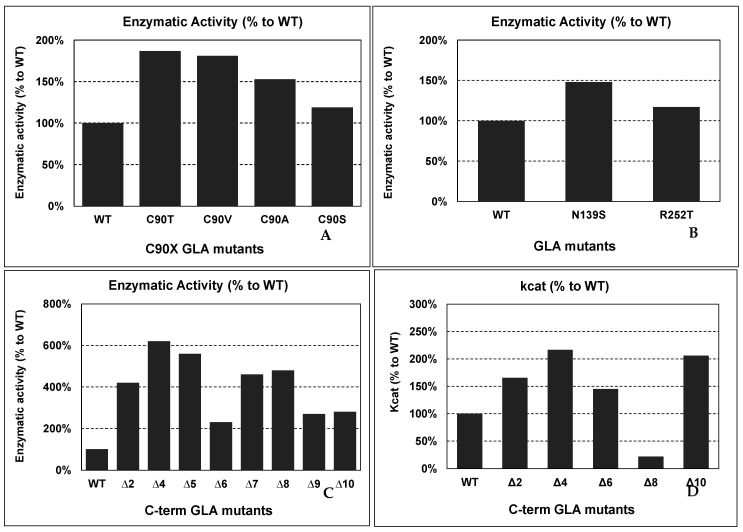
Enzymatic activity of several “improved” GLA mutants, compared to wild-type GLA enzyme. The mutants shown are examples described by several authors. Abbreviations: WT: wild-type GLA; ΔX: C-terminal deletions corresponding to “X” missing residues.

For example, Qiu et al. produced in vitro recombinant mutants of GLA with the free cysteines (C90 and C174) mutated to understand their role in the structure and function of the protein [37]. Mutations in cysteines involved in disulfide bond formation are common in FD patients, but mutations in free cysteines appear less frequently. C90 is more tolerant to changes than C174, due to its structural environment. Conservative mutations at C90 do not affect protein function and showed even higher enzymatic activities than the wild-type enzyme did. These mutations were C90T, C90V, C90A, and C90S, and they showed 187%, 181%, 153%, and 119% of enzymatic activity compared to wild-type GLA, respectively (see Figure 3 panel A). The explanation for the increased catalytic activity could be that subtle modifications induced by conservative modifications lead to an enhancement of the conformational flexibility.

Lukas et al. analyzed a significant number of mutations to establish a correlation with clinical manifestations and identify novel mutations [62]. In this study, two-point mutations (N139S and R252T) showed higher enzymatic activities (see Figure 3 panel B) than the wild-type enzyme (148% and 117%, respectively) did. However, N139S was related to clinical manifestations of the disease in a study carried out by Havndrup et al. [9]. The mutation was found in a woman with asymmetric septal hypertrophy, a significant left ventricular outflow tract gradient, and chronic obstructive pulmonary disease cardiac symptoms. N139S is part of the 138GNK140 sequence, a signature sequence in the family 27 of glycoside hydrolases, which could explain the pathogenesis of the mutation. The discrepancy between the higher activity and the presence of possible FD symptoms demands for further characterization of the N139S mutation.

Furthermore, carboxyl-terminal deletions have been reported to increase GLA enzymatic activity. Miyamura et al. produced in vitro mutants with various deletions of the C-terminus in COS-1 cells to analyze their effect [47]. They reported an increase in enzymatic activity when the deletion occurs in the last ten residues. In contrast, if the deletion comprises more than those ten residues, it results in a drastic loss of enzymatic activity. The GLA activities observed were 420%, 620%, 560%, 230%, 460%, 480%, 270%, and 280% of those of WT GLA for ∆2, ∆4, ∆5, ∆6, ∆7, ∆8, ∆9, and ∆10, respectively (see Figure 3 panel C).

Further characterization of the carboxyl-terminal truncations was carried out by Meghdari et al. [46]. They produced (in the methylotrophic yeast *Pichia Pastoris*) and purified the mutant enzymes with deletions of 2, 4, 6, 8, and 10 C-terminal amino acids (Δ2, Δ4, Δ6, Δ8, Δ10, respectively) to perform quantitative enzyme assays. The results showed increases in the kcat and Vmax with deletions of 2, 4, 6, and 10 amino acids (see Figure 3 panel D). However, the deletion of 8 C-term amino acids decreased the kcat. Discrepancies in this Δ8 mutant with the previous report from Miyamura could be explained due to experimental differences between them. Meghdari et al. used *P. Pastoris* as the expression system, while, in the previous experiment, COS-1 cells were used. Therefore, there could be differences in GLA post-translational modifications that specifically affect the protein activity/stability of this mutant in each expression system, as well as the presence of other proteins in the cytoplasm of *P. pastoris* or COS-1 cells that could interact with the protein, affecting its catalytic activity.

The final residues of each monomer in GLA do not have an associated electron density, and it is likely that it is a disordered region. Furthermore, there is a low sequence homology between GLA from different species. The C-terminus is located far from the active site, so mutations in this position do not directly affect the active site. Altogether, it can be hypothesized that the impact of C-terminus deletions on the activity of the enzyme could be due to alterations of the enzyme’s three-dimensional structure. This could influence enzyme dimerization, substrate binding, or potential interactions with other molecules in the cell.

#### Putative Applications of Improved Mutants in Innovative FD Therapies

As mentioned before, the current therapy to treat patients suffering from FD, and particularly those with mutations that are not responsive to PCT, is enzyme replacement therapy (ERT). The administration of recombinant GLA decreases Gb3 levels and alleviates clinical symptoms. However, the appearance of anti-drug antibodies (ADAs) against recombinant GLA can negatively influence the efficacy of the treatment by changing the distribution, cellular uptake, cellular localization, or catalytic activity of the administered enzyme [64]. Thus, further studies exploring the development of ADAs in FD patients using ERT and the potential impact of such antibodies on the efficacy of the therapy are necessary.

Vedder et al. reported that administering higher doses of the enzyme resulted in a stronger decrease in Gb3 with less impact of antibody formation [65]. Therefore, the use of mutated enzymes with increased activity would be expected to overcome the inhibitory effect of antibodies on treatment effectiveness. By using an enzyme that is more active on a per milligram basis, a therapeutic effect equivalent to the wild-type enzyme could be achievable through infusion of a lower dose. However, this approach is not being explored despite its apparent benefits. One possible explanation to this could be related to legal issues regarding the use of mutant enzymes instead of the wild-type enzyme, in terms of safety, side effects, and efficacy.

Nevertheless, mutants have already been approved for the treatment of other diseases, such as diabetes. Insulin lispro ([Lys(B28, Pro(B29)]-human insulin) is an insulin analogue in which the natural amino acid sequence of the B-chain at positions 28 and 29 is inverted, reducing potential dimerization of the molecule [66]. This allows larger amounts of active monomeric insulin to be available for postprandial injections. Insulin lispro, based on a mutation in the C-terminus, was first approved for use in the United States in 1996 and has proved to be efficient and safe.

As described above, removal of several residues from the C-terminal sequence of wild-type GLA results in a significant increase in enzymatic activity. Moreover, both agalsidase beta and agalsidase alfa display some degree of C-terminal heterogeneity with truncated species lacking either one or two C-terminal residues [67]. Agalsidase beta contains mainly full-length protein with 7.6% of Δ1 and 22.8% of Δ2, while agalsidase alfa contains only 5.7% full length, with 73.2% of Δ1 and 21.1% of Δ2. These differences most likely occur due to proteolytic processing of the mature full-length protein, according to the authors. The significantly increased activity of some of the mutants with C-terminal deletions suggests the starting point for an improved treatment for FD.

Furthermore, the existence of mutants with higher enzymatic activity raises the possibility of studying potential combinations of mutations that could lead to even higher activities. Therefore, further analysis and characterization of the mutations mentioned, as well as the investigation of novel mutations with similar properties, is required to determine the improvement that would mean the use of such mutants in ERT.

### 4.7. Other Modifications in Recombinant GLAs in Innovative FD Therapies

Other strategies to improve ERT have been studied, mainly regarding post-translational modifications, for example, the use of a recombinant GLA with enhanced sialylation obtained through in vitro glycosylation [68]. Sialic acid capping is essential for proper tissue targeting and increased half-life in serum as it masks the terminal galactose, preventing it from being recognized by the asialoglycoprotein receptor in the liver, which would end in clearance from the blood. Another strategy studied is covalent bonding between two GLA subunits. Ruderfer et al. studied this approach using PEG-based cross-linkers of various lengths and, currently, the developed enzyme (Pegunigalsidase alfa, by Protalix) is being tested in phase III of clinical trials [69]. These strategies could also be implemented with the use of improved mutants, which could result in a drug with increased stability and half-life in serum, as well as with the possibility to overcome the inhibitory effect of ADAs by applying lower doses of the enzyme.

## 5. Conclusions

Although important actions have been taken to improve the treatment of FD, a definitive cure is not yet available. An incomplete understanding of disease pathogenesis still limits the ability to effectively treat FD patients. ERT and PCT are currently approved for FD; however, these treatments are not curative and show several limitations. Further study of the mechanisms involved in the disease will allow the development of new therapies, as well as the improvement of the existing ones. The current availability of animal models together with the study and characterization of GLA mutants can be used as tools for the purpose.

**Figure 4:**
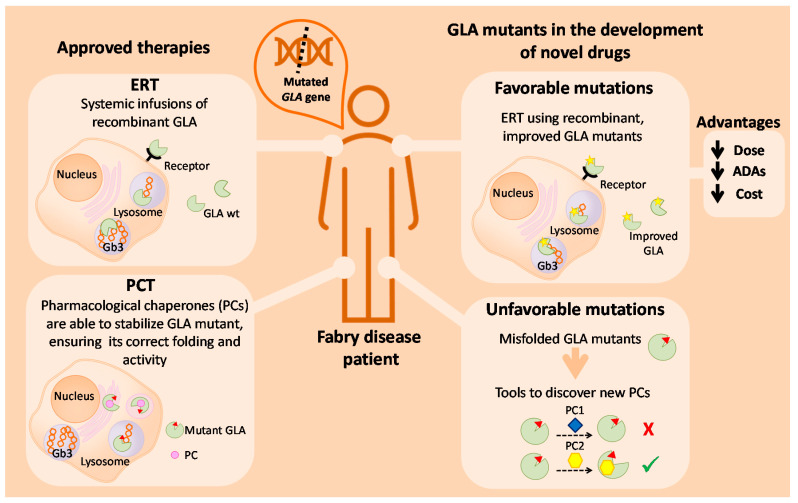
Schematic overview of current therapies for FD treatment, and the putative use of GLA enzyme mutants as tools to develop new therapeutic approaches for FD.

In Figure 4, we summarize the putative use of GLA mutants as a tool for the study and development of novel drugs for the treatment of FD. For a small subset of patients with specific amenable mutations, treatment with PCs might be a suitable approach. Improvements in this therapy can be assessed by making use of unfavorable mutants with incorrect folding. Inhibitors such as DGJ (Migalastat) are not the ideal drugs, as they cannot fully revert the phenotype of the disease. Therefore, the screening of new molecules and in vitro analysis with GLA mutants could lead to the discovery of novel PCs for the treatment of FD. Concerning this matter, a useful approach is drug repositioning as it allows the lowering of the costs and shortening of the duration of the approval process. For the remaining patients, ERT is still the main treatment of the disease. It is not genotype-dependent, as PCT, but presents other limitations that influence its effectiveness. One of the main challenges with ERT (apart from targeted, specific delivery of GLA to difficult sites of pathology such as the kidney and heart in FD, infusion-associated reactions, and that it is a lifelong therapy requiring intravenous administration every 2 weeks associated with a high cost) is the associated formation of anti-drug antibodies (ADAs) against recombinant GLA. The use of mutated enzymes with increased activity could overcome the inhibitory effect of antibodies by achieving the same (or even higher) therapeutic effect with lower GLA doses. Such lower doses of infused enzyme would probably result in lower or reduced ADA formation. In this sense, the relationship between the amounts of infused GLA and ADAs titer has already been published—Vedder et al. reported that antibodies were more frequent in patients treated with agalsidase beta at 1.0 mg/kg than in patients treated with agalsidase alfa at 0.2 mg/kg (*p* = 0.005) [65]. Thus, GLA mutations involving C-terminal deletions seem to be a very promising approach to obtain “improved” versions of the therapeutic enzyme, allowing a similar therapeutic outcome with lower amounts of infused enzyme.

Enzymatic activity is not the only factor to take into account in order to obtain improved therapeutic enzymes. Other characteristics such as proper biodistribution and pharmacokinetics or plasma stability, among others, are key to achieve better therapeutic effects. Such parameters should be deeply characterized for every mutant, apart from their specific enzymatic activity. For example, Qui et al. showed that mutants C90S, C174S, and C90S/C174S are enzymatically active, structurally intact, and thermodynamically stable, as measured by circular dichroism and thermal denaturation [37]. In another study, C-terminal deletions of the GLA enzyme showed not only better enzymatic performance but also an equal thermal stability at 30 °C, 40 °C, and 50 °C for wild-type and C-terminal GLA mutants [46]. These studies show that researchers in the field are fully aware of the importance of other parameters than only enzymatic activity to check new putative candidates for ERT in FD.

Different approaches to improve ERT are being investigated. Therefore, the combination of these strategies with the exploitation of favorable mutations could lead to a recombinant GLA enzyme with much higher activity and stability. One concern about the use of GLA mutants as drugs for FD treatment is their approval by regulatory authorities. However, it has great potential to confer clinical benefits and could mean an improvement in the current methods. Therefore, it may be worth investing more time and efforts, despite the inconveniences, with the aim of achieving better results. Further investigation should be addressed to prove its safety and effectiveness. Our understanding of the pathogenesis of the disease is constantly changing and we should be ready to implement possible innovations.

In the long-term, other strategies can be implemented, such as gene therapy. However, although a promising alternative, it still needs additional development. Therefore, for the moment, investing in improvements of current therapies is a more achievable option to develop new tools to treat FD patients.

## References

[1] Germain D.P. (2010). Fabry disease. Orphanet J. Rare Dis..

[2] Bernardes T.P., Foresto R.D., Kirsztajn G.M. (2020). Fabry disease: Genetics, pathology, and treatment. Rev. Assoc. Med. Bras..

[3] Stenson P.D., Ball E.V., Mort M., Phillips A.D., Shiel J.A., Thomas N.S.T., Abeysinghe S., Krawczak M., Cooper D.N. (2003). Human Gene Mutation Database (HGMD^®^): 2003 update: HGMD 2003 UPDATE. Hum. Mutat..

[4] Simonetta I., Tuttolomondo A., Di Chiara T., Miceli S., Vogiatzis D., Corpora F., Pinto A. (2018). Genetics and Gene Therapy of Anderson-Fabry Disease. Curr. Gene Ther..

[5] Sakuraba H., Oshima A., Fukuhara Y., Shimmoto M., Nagao Y., Bishop D.F., Desnick R.J., Suzuki Y. (1990). Identification of point mutations in the alpha-galactosidase A gene in classical and atypical hemizygotes with Fabry disease. Am. J. Hum. Genet..

[6] Andrade J., Waters P.J., Singh R.S., Levin A., Toh B.-C., Vallance H.D., Sirrs S. (2008). Screening for Fabry disease in patients with chronic kidney disease: Limitations of plasma alpha-galactosidase assay as a screening test. Clin. J. Am. Soc. Nephrol..

[7] Beck M. (2006). The Mainz Severity Score Index (MSSI): Development and validation of a system for scoring the signs and symptoms of Fabry disease. Acta Paediatr..

[8] Miller J.J., Kanack A.J., Dahms N.M. (2020). Progress in the understanding and treatment of Fabry disease. Biochim. Biophys. Acta Gen. Subj..

[9] Havndrup O., Christiansen M., Stoevring B., Jensen M., Hoffman-Bang J., Andersen P.S., Hasholt L., Nørremølle A., Feldt-Rasmussen U., Køber L. (2010). Fabry disease mimicking hypertrophic cardiomyopathy: Genetic screening needed for establishing the diagnosis in women. Eur. J. Heart Fail..

[10] Aerts J.M., Groener J.E., Kuiper S., Donker-Koopman W.E., Strijland A., Ottenhoff R., van Roomen C., Mirzaian M., Wijburg F.A., Linthorst G.E. (2008). Elevated globotriaosylsphingosine is a hallmark of Fabry disease. Proc. Natl. Acad. Sci. USA.

[11] Bichet D.G., Aerts J.M., Auray-Blais C., Maruyama H., Mehta A.B., Skuban N., Krusinska E., Schiffmann R. (2021). Assessment of plasma lyso-Gb(3) for clinical monitoring of treatment response in migalastat-treated patients with Fabry disease. Genet. Med..

[12] Arends M., Biegstraaten M., Hughes D.A., Mehta A., Elliott P.M., Oder D., Watkinson O.T., Vaz F.M., van Kuilenburg A.B.P., Wanner C. (2017). Retrospective study of long-term outcomes of enzyme replacement therapy in Fabry disease: Analysis of prognostic factors. PLoS ONE.

[13] Cammarata G., Scalia S., Colomba P., Zizzo C., Pisani A., Riccio E., Montalbano M., Alessandro R., Giordano A., Duro G. (2018). A pilot study of circulating microRNAs as potential biomarkers of Fabry disease. Oncotarget.

[14] Boutin M., Auray-Blais C. (2015). Metabolomic Discovery of Novel Urinary Galabiosylceramide Analogs as Fabry Disease Biomarkers. J. Am. Soc. Mass Spectrom..

[15] Matafora V., Cuccurullo M., Beneduci A., Petrazzuolo O., Simeone A., Anastasio P., Mignani R., Feriozzi S., Pisani A., Comotti C. (2015). Early markers of Fabry disease revealed by proteomics. Mol. Biosyst..

[16] Bishop D.F., Kornreich R., Desnick R.J. (1988). Structural organization of the human alpha-galactosidase A gene: Further evidence for the absence of a 3’ untranslated region. Proc. Natl. Acad. Sci. USA.

[17] Garman S.C., Garboczi D.N. (2004). The molecular defect leading to Fabry disease: Structure of human alpha-galactosidase. J. Mol. Biol..

[18] Ioannou Y.A., Zeidner K.M., Grace M.E., Desnick R.J. (1998). Human alpha-galactosidase A: Glycosylation site 3 is essential for enzyme solubility. Biochem. J..

[19] Matsuura F., Ohta M., Ioannou Y.A., Desnick R.J. (1998). Human -galactosidase A: Characterization of the N-linked oligosaccharides on the intracellular and secreted glycoforms overexpressed by Chinese hamster ovary cells. Glycobiology.

[20] Poole L.B. (2015). The basics of thiols and cysteines in redox biology and chemistry. Free Radic. Biol. Med..

[21] Nakaniwa T., Fukada H., Inoue T., Gouda M., Nakai R., Kirii Y., Adachi M., Tamada T., Segawa S., Kuroki R. (2012). Seven Cysteine-Deficient Mutants Depict the Interplay between Thermal and Chemical Stabilities of Individual Cysteine Residues in Mitogen-Activated Protein Kinase c-Jun N-Terminal Kinase 1. Biochemistry.

[22] Saftig P., Mehta A., Beck M., Sunder-Plassmann G. (2006). Physiology of the lysosome. Fabry Disease: Perspectives from 5 Years of FOS.

[23] Koshland D.E. (1953). Stereochemistry and the Mechanism of Enzymatic Reactions. Biol. Rev..

[24] Guce A.I., Clark N.E., Salgado E.N., Ivanen D.R., Kulminskaya A.A., Brumer H., Garman S.C. (2010). Catalytic Mechanism of Human α-Galactosidase. J. Biol. Chem..

[25] Ohashi T. (2019). Current status and future prospect of enzyme replacement therapy for Fabry disease. Rinsho Shinkeigaku.

[26] Stappers F., Scharnetzki D., Schmitz B., Manikowski D., Brand S., Grobe K., Lenders M., Brand E. (2020). Neutralising anti-drug antibodies in Fabry disease can inhibit endothelial enzyme uptake and activity. J. Inherit. Metab. Dis..

[27] Liguori L., Monticelli M., Allocca M., Hay Mele B., Lukas J., Cubellis M.V., Andreotti G. (2020). Pharmacological Chaperones: A Therapeutic Approach for Diseases Caused by Destabilizing Missense Mutations. Int. J. Mol. Sci..

[28] Ishii S., Chang H.-H., Kawasaki K., Yasuda K., Wu H.-L., Garman S.C., Fan J.-Q. (2007). Mutant α-galactosidase A enzymes identified in Fabry disease patients with residual enzyme activity: Biochemical characterization and restoration of normal intracellular processing by 1-deoxygalactonojirimycin. Biochem. J..

[29] Lenders M., Stappers F., Brand E. (2020). In Vitro and In Vivo Amenability to Migalastat in Fabry Disease. Mol. Ther. Methods Clin. Dev..

[30] Okumiya T., Ishii S., Takenaka T., Kase R., Kamei S., Sakuraba H., Suzuki Y. (1995). Galactose Stabilizes Various Missense Mutants of α-Galactosidase in Fabry Disease. Biochem. Biophys. Res. Commun..

[31] Fan J.-Q., Ishii S., Asano N., Suzuki Y. (1999). Accelerated transport and maturation of lysosomal α–galactosidase A in Fabry lymphoblasts by an enzyme inhibitor. Nat. Med..

[32] Asano N., Ishii S., Ikeda K., Kizu H., Yasuda K., Kato A., Martin O.R., Fan J.-Q. (2000). *In vitro* inhibition and intracellular enhancement of lysosomal α-galactosidase A activity in Fabry lymphoblasts by 1-deoxygalactonojirimycin and its derivatives: Enhancement of α-Gal A in Fabry lymphoblasts. Eur. J. Biochem..

[33] Guce A.I., Clark N.E., Rogich J.J., Garman S.C. (2011). The Molecular Basis of Pharmacological Chaperoning in Human α-Galactosidase. Chem. Biol..

[34] van der Tol L., Smid B.E., Poorthuis B.J.H.M., Biegstraaten M., Deprez R.H.L., Linthorst G.E., Hollak C.E.M. (2014). A systematic review on screening for Fabry disease: Prevalence of individuals with genetic variants of unknown significance. J. Med. Genet..

[35] Mehta A., Hughes D.A., Adam M.P., Ardinger H.H., Pagon R.A., Wallace S.E., Bean L.J.H., Stephens K., Amemiya A. (1993). Fabry Disease. GeneReviews^®^.

[36] Germain D.P., Poenaru L. (1999). Fabry Disease: Identification of Novel Alpha-Galactosidase A Mutations and Molecular Carrier Detection by Use of Fluorescent Chemical Cleavage of Mismatches. Biochem. Biophys. Res. Commun..

[37] Qiu H., Honey D.M., Kingsbury J.S., Park A., Boudanova E., Wei R.R., Pan C.Q., Edmunds T. (2015). Impact of cysteine variants on the structure, activity, and stability of recombinant human α-galactosidase A: Cysteine Variants of Human α-Galactosidase A. Protein Sci..

[38] Lee B.H., Heo S.H., Kim G.-H., Park J.-Y., Kim W.-S., Kang D.-H., Choe K.H., Kim W.-H., Yang S.H., Yoo H.-W. (2010). Mutations of the GLA gene in Korean patients with Fabry disease and frequency of the E66Q allele as a functional variant in Korean newborns. J. Hum. Genet..

[39] Serebrinsky G., Calvo M., Fernandez S., Saito S., Ohno K., Wallace E., Warnock D., Sakuraba H., Politei J. (2015). Late onset variants in Fabry disease: Results in high risk population screenings in Argentina. Mol. Genet. Metab. Rep..

[40] Meng Y., Zhang W.-M., Shi H.-P., Wei M., Huang S.-Z. (2010). Clinical manifestations and mutation study in 16 Chinese patients with Fabry disease. Zhonghua Yi Xue Za Zhi.

[41] Garman S.C., Garboczi D.N. (2002). Structural basis of Fabry disease. Mol. Genet. Metab..

[42] Guffon N., Froissart R., Chevalier-Porst F., Maire I. (1998). Mutation analysis in 11 French patients with Fabry disease. Hum. Mutat..

[43] Ebrahim H.Y., Baker R.J., Mehta A.B., Hughes D.A. (2012). Functional analysis of variant lysosomal acid glycosidases of Anderson-Fabry and Pompe disease in a human embryonic kidney epithelial cell line (HEK 293 T). J. Inherit. Metab. Dis..

[44] Nakao S., Takenaka T., Maeda M., Kodama C., Tanaka A., Tahara M., Yoshida A., Kuriyama M., Hayashibe H., Sakuraba H. (1995). An Atypical Variant of Fabry’s Disease in Men with Left Ventricular Hypertrophy. N. Engl. J. Med..

[45] Germain D.P., Brand E., Burlina A., Cecchi F., Garman S.C., Kempf J., Laney D.A., Linhart A., Maródi L., Nicholls K. (2018). Phenotypic characteristics of the p.Asn215Ser (p.N215S) *GLA* mutation in male and female patients with Fabry disease: A multicenter Fabry Registry study. Mol. Genet. Genom. Med..

[46] Meghdari M., Gao N., Abdullahi A., Stokes E., Calhoun D.H. (2015). Carboxyl-Terminal Truncations Alter the Activity of the Human α-Galactosidase A. PLoS ONE.

[47] Miyamura N., Araki E., Matsuda K., Yoshimura R., Furukawa N., Tsuruzoe K., Shirotani T., Kishikawa H., Yamaguchi K., Shichiri M. (1996). A carboxy-terminal truncation of human alpha-galactosidase A in a heterozygous female with Fabry disease and modification of the enzymatic activity by the carboxy-terminal domain. Increased, reduced, or absent enzyme activity depending on number of amino. J. Clin. Investig..

[48] Garman S.C. (2007). Structure-function relationships in α-galactosidase A: Structure-function relationships in α-galactosidase A. Acta Paediatr..

[49] Wu X., Katz E., Valle M.C.D., Mascioli K., Flanagan J.J., Castelli J.P., Schiffmann R., Boudes P., Lockhart D.J., Valenzano K.J. (2011). A pharmacogenetic approach to identify mutant forms of α-galactosidase a that respond to a pharmacological chaperone for Fabry disease. Hum. Mutat..

[50] Citro V., Cammisa M., Liguori L., Cimmaruta C., Lukas J., Cubellis M., Andreotti G. (2016). The Large Phenotypic Spectrum of Fabry Disease Requires Graduated Diagnosis and Personalized Therapy: A Meta-Analysis Can Help to Differentiate Missense Mutations. Int. J. Mol. Sci..

[51] Yu Y., Mena-Barragán T., Higaki K., Johnson J.L., Drury J.E., Lieberman R.L., Nakasone N., Ninomiya H., Tsukimura T., Sakuraba H. (2014). Molecular Basis of 1-Deoxygalactonojirimycin Arylthiourea Binding to Human α-Galactosidase A: Pharmacological Chaperoning Efficacy on Fabry Disease Mutants. ACS Chem. Biol..

[52] Mena-Barragán T., Narita A., Matias D., Tiscornia G., Nanba E., Ohno K., Suzuki Y., Higaki K., Garcia Fernández J.M., Ortiz Mellet C. (2015). pH-Responsive Pharmacological Chaperones for Rescuing Mutant Glycosidases. Angew. Chem. Int. Ed..

[53] Citro V., Peña-García J., den-Haan H., Pérez-Sánchez H., Del Prete R., Liguori L., Cimmaruta C., Lukas J., Cubellis M.V., Andreotti G. (2016). Identification of an Allosteric Binding Site on Human Lysosomal Alpha-Galactosidase Opens the Way to New Pharmacological Chaperones for Fabry Disease. PLoS ONE.

[54] Hay Mele B., Citro V., Andreotti G., Cubellis M.V. (2015). Drug repositioning can accelerate discovery of pharmacological chaperones. Orphanet J. Rare Dis..

[55] Rigat B., Mahuran D. (2009). Diltiazem, a L-type Ca^2+^ channel blocker, also acts as a pharmacological chaperone in Gaucher patient cells. Mol. Genet. Metab..

[56] Lukas J., Pockrandt A.-M., Seemann S., Sharif M., Runge F., Pohlers S., Zheng C., Gläser A., Beller M., Rolfs A. (2015). Enzyme Enhancers for the Treatment of Fabry and Pompe Disease. Mol. Ther..

[57] Maegawa G.H.B., Tropak M.B., Buttner J.D., Rigat B.A., Fuller M., Pandit D., Tang L., Kornhaber G.J., Hamuro Y., Clarke J.T.R. (2009). Identification and Characterization of Ambroxol as an Enzyme Enhancement Agent for Gaucher Disease. J. Biol. Chem..

[58] Calamini B., Silva M.C., Madoux F., Hutt D.M., Khanna S., Chalfant M.A., Saldanha S.A., Hodder P., Tait B.D., Garza D. (2012). Small-molecule proteostasis regulators for protein conformational diseases. Nat. Chem. Biol..

[59] Muntau A.C., Leandro J., Staudigl M., Mayer F., Gersting S.W. (2014). Innovative strategies to treat protein misfolding in inborn errors of metabolism: Pharmacological chaperones and proteostasis regulators. J. Inherit. Metab. Dis..

[60] Seemann S., Ernst M., Cimmaruta C., Struckmann S., Cozma C., Koczan D., Knospe A.-M., Haake L.R., Citro V., Bräuer A.U. (2020). Proteostasis regulators modulate proteasomal activity and gene expression to attenuate multiple phenotypes in Fabry disease. Biochem. J..

[61] Mu T.-W., Ong D.S.T., Wang Y.-J., Balch W.E., Yates J.R., Segatori L., Kelly J.W. (2008). Chemical and Biological Approaches Synergize to Ameliorate Protein-Folding Diseases. Cell.

[62] Lukas J., Knospe A.-M., Seemann S., Citro V., Cubellis M.V., Rolfs A. (2017). In Vitro Enzyme Measurement to Test Pharmacological Chaperone Responsiveness in Fabry and Pompe Disease. J. Vis. Exp..

[63] Cammisa M., Correra A., Andreotti G., Cubellis M. (2013). Fabry_CEP: A tool to identify Fabry mutations responsive to pharmacological chaperones. Orphanet J. Rare Dis..

[64] Deegan P.B. (2012). Fabry disease, enzyme replacement therapy and the significance of antibody responses. J. Inherit. Metab. Dis..

[65] Vedder A.C., Breunig F., Donker-Koopman W.E., Mills K., Young E., Winchester B., Ten Berge I.J.M., Groener J.E.M., Aerts J.M.F.G., Wanner C. (2008). Treatment of Fabry disease with different dosing regimens of agalsidase: Effects on antibody formation and GL-3. Mol. Genet. Metab..

[66] Howey D.C., Bowsher R.R., Brunelle R.L., Woodworth J.R. (1994). [Lys(B28), Pro(B29)]-Human Insulin: A Rapidly Absorbed Analogue of Human Insulin. Diabetes.

[67] Lee K., Jin X., Zhang K., Copertino L., Andrews L., Baker-Malcolm J., Geagan L., Qiu H., Seiger K., Barngrover D. (2003). A biochemical and pharmacological comparison of enzyme replacement therapies for the glycolipid storage disorder Fabry disease. Glycobiology.

[68] Sohn Y., Lee J.M., Park H.-R., Jung S.-C., Park T.H., Oh D.-B. (2013). Enhanced sialylation and in vivo efficacy of recombinant human α-galactosidase through in vitro glycosylation. BMB Rep..

[69] Ruderfer I., Shulman A., Kizhner T., Azulay Y., Nataf Y., Tekoah Y., Shaaltiel Y. (2018). Development and Analytical Characterization of Pegunigalsidase Alfa, a Chemically Cross-Linked Plant Recombinant Human α-Galactosidase-A for Treatment of Fabry Disease. Bioconjug. Chem..

